# Abiotic and Biotic Degradation of Oxo-Biodegradable Plastic Bags by *Pleurotus ostreatus*

**DOI:** 10.1371/journal.pone.0107438

**Published:** 2014-11-24

**Authors:** José Maria Rodrigues da Luz, Sirlaine Albino Paes, Denise Mara Soares Bazzolli, Marcos Rogério Tótola, Antônio Jacinto Demuner, Maria Catarina Megumi Kasuya

**Affiliations:** Departamento de Microbiologia, Universidade Federal de Viçosa, Viçosa, Minas Gerais, Brazil; Nanyang Technological University, Singapore

## Abstract

In this study, we evaluated the growth of *Pleurotus ostreatus* PLO6 using oxo-biodegradable plastics as a carbon and energy source. Oxo-biodegradable polymers contain pro-oxidants that accelerate their physical and biological degradation. These polymers were developed to decrease the accumulation of plastic waste in landfills. To study the degradation of the plastic polymers, oxo-biodegradable plastic bags were exposed to sunlight for up to 120 days, and fragments of these bags were used as substrates for *P. ostreatus*. We observed that physical treatment alone was not sufficient to initiate degradation. Instead, mechanical modifications and reduced titanium oxide (TiO_2_) concentrations caused by sunlight exposure triggered microbial degradation. The low specificity of lignocellulolytic enzymes and presence of endomycotic nitrogen-fixing microorganisms were also contributing factors in this process.

## Introduction

Several Brazilian cities have banned the distribution of plastic bags by supermarkets and other commercial establishments because plastic bags can take a long time to degrade in dumps and landfills. As an alternative, oxo-biodegradable plastics (D2W) have been recommended.

The biodegradability of a polymer is determined by its chemical structure and morphology [1]. Polyethylene, due to its high hydrophobicity and long carbon chains, is very resistant to biodegradation [1],[2]. Under normal conditions, mineralization of this polymer takes more than 100 years [3]. In contrast to these petroleum-derived polymers, biodegradable polymers are cleaved by non-enzymatic processes (e.g., chemical hydrolysis, photolysis or pyrolysis) and subsequently degraded by algal, bacterial and fungal enzymes [4].

Oxo-biodegradable polymers contain pro-oxidants and pro-degrading compounds [5],[6] that are incorporated into the polymer chain to accelerate photo- or thermo-oxidation [5]–[7]. These pro-oxidants are metal ions or oxides, such as titanium oxide, that catalyze photo- or thermo-oxidation of the polymer [1],[5]–[8]. During photo-degradation, also termed pro-oxidant photocatalytic oxidation, free radicals produced of the reactions catalized by pro-oxidant causes scission in the polymer chain [6]–[8], facilitating microbial degradation [4],[7]. UV light serves as a catalyst for this photocatalytic oxidation process [8]. Da Luz et al. [9] showed that *Pleurotus ostreatus* PLO6 degrades substrates containing oxo-biodegradable polyethylene, albeit at a slow rate. However, this study did not pretreat samples with UV irradiation or heat and only evaluated degradation after 45 days. The aim of our study was to evaluate the abiotic and biotic degradation of oxo-biodegradable plastic bags throughout 120 days of sunlight exposure and 90 days of fungal growth. We also analyzed the presence of endomycotic nitrogen-fixing microorganisms on hyphae *of P. ostreatus* that can help in fungal growth in substrate containing low concentration nitrogen.

## Materials and Methods

Oxo-biodegradable plastic bags were kindly donated by Fundação Arthur Bernandes, Universidade Federal de Viçosa, MG, Brazil. The identification of pro-oxidants and polymers was confirmed by scanning electron microscopy (SEM) coupled with X-ray diffraction and Fourier transform infrared spectroscopy (FTIR).

*Pleurotus ostreatus* PLO6 (GenBank accession number KC782771) used in this study belongs to the fungal collection of the Department of Microbiology of Universidade Federal de Viçosa. The stock cultures were maintained on potato dextrose agar (PDA) at 25°C. After 15 days, mycelial disks were punched out with a 7 mm cork borer and used to inoculate the substrates.

### 2.1 Abiotic degradation

The oxo-biodegradable plastic bags were exposed to sunlight for 30, 60, 90 or 120 days. The samples were placed every 30 days and after 120 days of sunlight exposure all samples were removed. This procedure took place during the summer at a site protected from rainwater.

To evaluate the effect of sunlight, the physical and chemical alterations in the plastic were analyzed (see item 2.4). A control treatment was also prepared using plastic bags that were not exposed to sunlight.

### 2.2 Biotic degradation

Plastic bags with or without sunlight exposure were cut into fragments (5 cm×1 cm), and 10 g of this material was placed into a 100 mL glass flask containing 0.1 g of a commercially available paper towel [9], so the carbon source consisted mainly by the plastic. Five milliliters of mineral medium [7],[10], supplemented with filter-sterilized thiamine-HCl, was added. Each flask was inoculated with four agar discs (6–8 mm) containing mycelium of *P. ostreatus* and incubated at 25°C for 30, 60 or 90 days.

### 2.3 Fungal respiratory activity

Respiratory activity was measured by attaching the flasks to a continuous-flow respirometer coupled with an infrared CO_2_ detector (TR-RM8 Respirometer Multiplexer, Sable Systems). CO_2_ measurements were carried out every 24 h [11], and flasks remained attached to the respirometer for 90 days.

### 2.4 Analysis of the biodegradation of oxo-biodegradable polymers

Physical changes, such as the formation of pits and cracks, and fungal colonization on the plastic surface were analyzed by SEM (Leo, 1430VP) at 5000 x magnification [5],[9],[12]. Mechanical alterations of the plastic were analyzed by universal testing equipment (Instron model 3367). Chemical changes, such as the disappearance or appearance of new functional groups and bond scissions, were analyzed by FTIR [6],[7],[13].

To determinate the dry mass, the flasks containing fungal mycelium and substrates were dried at 105°C until a constant weight was obtained.

### 2.5 Confirmation of the presence of endomycotic microorganisms

Total DNA of *P. ostreatus* PLO6 was extracted using an Ultra Clean^TM^ Power Soil kit (Mobio Laboratories, Solana Beach, CA, USA) according to the manufacturer's instructions. The DNA was used as a template to amplify the *nifH* gene, which is involved in the nitrogen fixation process. Primers 19F and 407R were used to amplify a 390 bp fragment of *nifH*, as described by Ueda et al. [14]. This step was followed by nested PCR using the oligonucleotide 19F-C (with addition of a GC-clamp) and the primer 278R [15], and yielded a 260 bp fragment. The PCR mixture contained 20 ng of total DNA, 0.2 µM of each oligonucleotide, 200 µM dNTP, 2 mM MgCl_2_, 0.5 mg mL^−1^ bovine serum albumin and 1.25 units of GO Taq DNA polymerase (Promega, Madison, USA) in a total reaction volume of 50 µL. PCR cycling conditions were described by Direito and Teixeira [15].

DNA fragments obtained by nested PCR were analyzed by DGGE (Model *DCode Systems*, BIO-RAD, California) [16]. DNA from the following diazotrophic bacteria was used as external markers: *Pseudomonas fluorescens* (ATCC 14), *Bradyrhizobium elkanii* (BR96), *Bradyrhizobium japonicum* (BR111), *Rhizobium tropici*, *Burkholderia cepacia*, *Burkholderia brasiliensis* (BR11340), *Burkholderia sabiae* (BR3405), *Paenibacillus validus*, *Stenotrophomonas maltrophila*, *Bacillus cereus* (ATCC 3) and *Acinetobacter calcoaceticus* (Figure S1). Twenty µL nested PCR samples containing 150 to 200 ng of DNA were loaded onto an 8% denatured polyacrylamide gradient gel (w/v) in 1 X Tris-acetate-EDTA (TAE) buffer. The denaturing gradient of the gel ranged from 50% to 65%, where 100% indicates a concentration of seven mol L^−1^ urea and 40% formamide. The gel was subjected to vertical electrophoresis for 12 h at 60 V and 60°C, stained for 40 min with *SYBR Gold* (1x) (Molecular Probes, Leiden, The Netherlands) and photographed under UV light with a Molecular Imaging system (Loccus Biotecnologic L-Pix Chemi, São Paulo, Brazil). Desired bands were excised, eluted and subjected to PCR with the same primers used previously but lacking the GC clamp. PCR products were sequenced by Macrogen, Inc. (Korea). All sequences were edited using Sequencher software (Version 4.1, Genes Dodes, MI; USA). The results were compared to sequences deposited in NCBI GenBank using the BLASTx tool.

### 2.6 Statistical analyses

The experiment followed a completely randomized design with five replicates. The data were subjected to variance analysis (ANOVA), and mean values were compared using Tukey's test or regression analysis (p<0.05) by Saeg software (version 9.1, Universidade Federal de Viçosa). The Pearson correlation coefficient was calculated between respiratory activity and the dry mass of the substrates.

## Results

### 3.1 Abiotic degradation

Titanium was the main mineral found in the oxo-biodegradable plastics (Table 1). It was also observed relatively high concentration of copper, zinc and silicon and low relative concentration of nitrogen (Table 1). Other elements essential to microbial metabolism, such as iron and manganese, were also present. Dyes, commonly used in producing commercial plastic bags, may be the source of these elements.

**Table 1:** Chemical composition of the oxo-biodegradable plastic before and after 120 days of sunlight exposure.

Mineral composition (weight %)*	Exposure time to sunlight (days)	
	0	120
Si	9.402±0.884 B	14.073±1.368 A
Ti	57.847±1.206 A	52.489±1.102 B
Mn	1.646±0.575 A	2.411±0.586 A
Fe	1.027±0.456 B	2.113±0.348 A
Co	1.494±0.469 A	1.773±0.451 A
Cu	14.974±0.985 A	12.087±0.950 B
Zn	11.036±0.966 A	11.025±0.865 A
Cd	2.055±0.357 B	3.385±0.264 A
N	0.565±0.082 A	0.641±0.071 A

*Percentage of relative concentration of the elements analyzed on the surface of the oxo-biodegradable plastic by scanning electron microscopy coupled with X-ray diffraction (semi-quantitative method). The mean (designated by different letters within the same line) differ by variance analysis (ANOVA) and Tukey's test (p<0.05).

**Figure 1:**
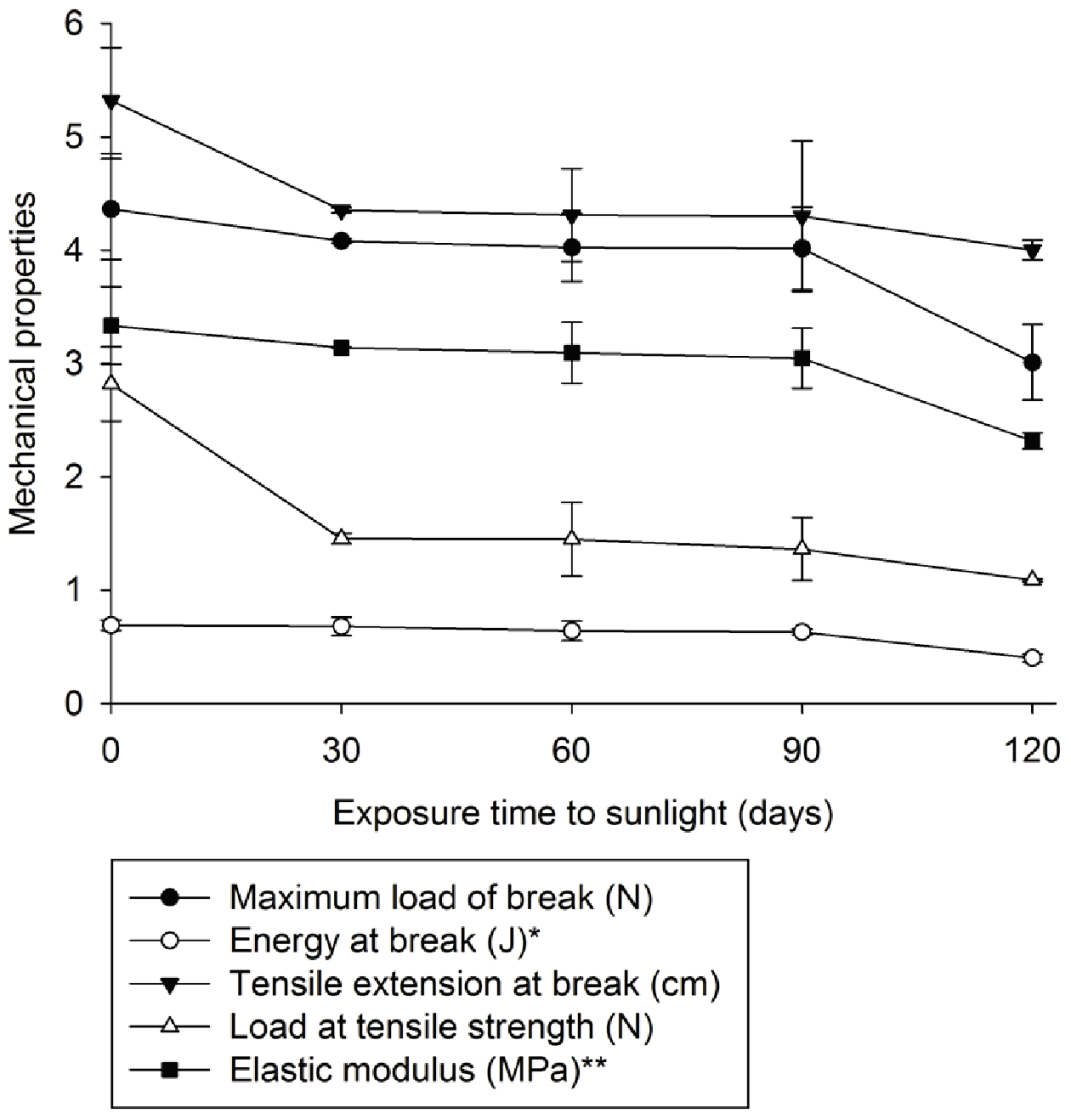
Mechanical properties of the oxo-biodegradable plastic before and after 30, 60, 90 or 120 days of sunlight exposure. *Value was multiplied by 10, ** Value was divided by 10.

Exposure to sunlight influenced the chemical composition (Table 1) and mechanical properties (Figure 1). It caused a decrease in titanium and copper concentrations and an increase in the concentrations of others elements (Table 1).

Sunlight predominantly affected the mechanical properties of the plastic at the beginning and end of exposure (Figure 1). After 120 days, the energy required to cause ruptures in the plastic was reduced. Elasticity and flexibility were similarly decreased (Figure 1).

**Figure 2:**
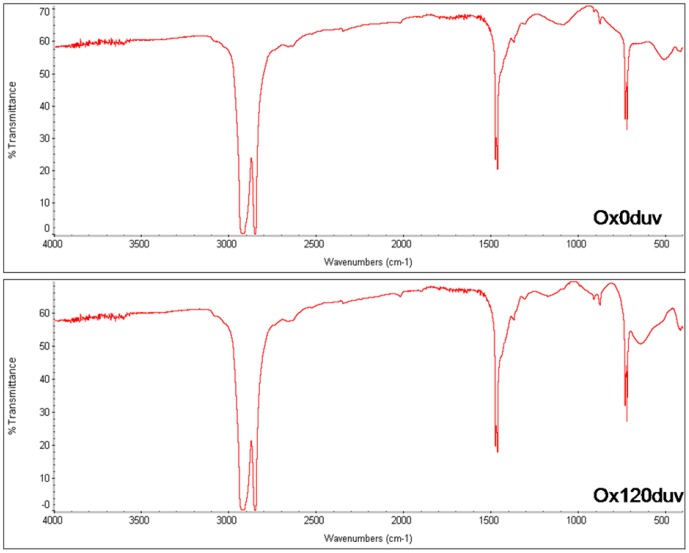
Spectrum of Fourier transform infrared spectroscopy of oxo-biodegradable plastic before (Ox0duv) and after 120 days of sunlight exposure (0x120duv).

**Figure 3:**
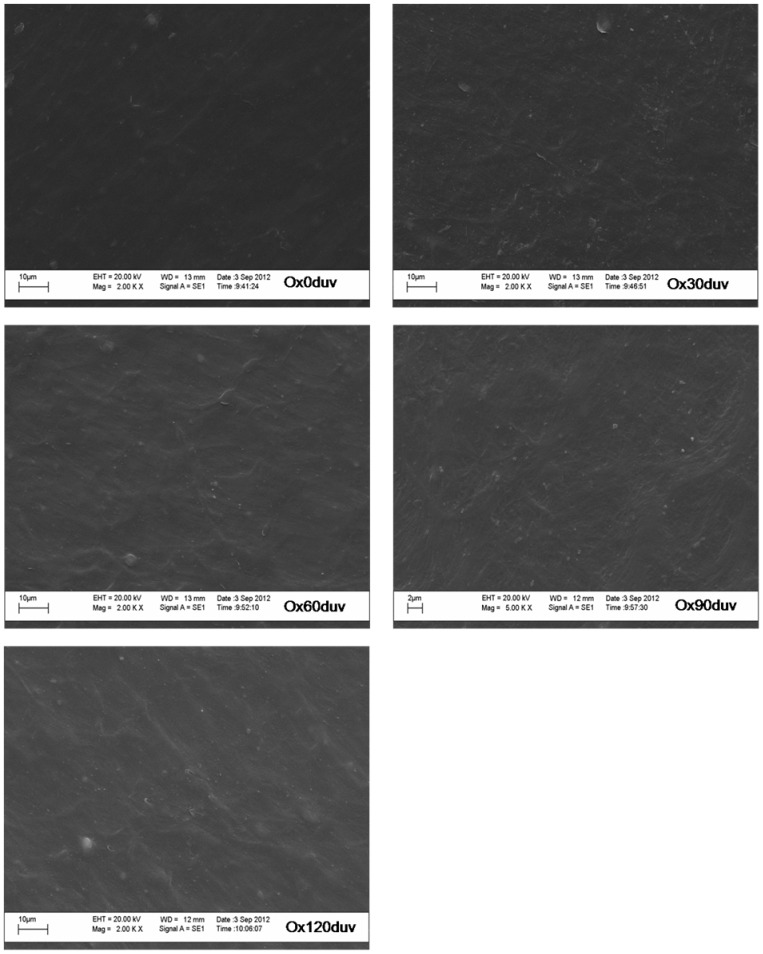
Scanning electron microscopy of oxo-biodegradable plastic before (Ox0duv) and after 30 (Ox30duv), 60 (Ox60duv), 90 (Ox90duv) and 120 days (Ox120duv) of sunlight exposure.

The chemical structure of polyethylene was unaffected by sunlight exposure (Figure 2). According to the manufacturer, the polymer chain is cleaved when the oxo-biodegradable polyethylene is exposed to the ultraviolet light, inducing photocatalytic oxidation by the pro-oxidant. The reduced titanium oxide concentration on plastic surface (Table 1) suggests the occurrence of TiO_2_ photocatalytic oxidation. However, this oxidation did not release enough energy to cause cleavage of the polymer chain (Figure 3).

Sunlight did not cause the formation of pits, cracks or breaks in the oxo-biodegradable plastic (Figure 3). However, the plastic became more translucent (Figure 3). Degradation of dyes and/or changes in other mechanical properties may have caused these modifications (Figure 1).

Our data shows that 120 days of sunlight was not sufficient to initiate abiotic degradation within the plastic. However, reduced titanium concentrations and modified mechanical properties may facilitate microbial attack, as claimed by the manufacturer.

### 3.2 Biotic degradation

**Figure 4:**
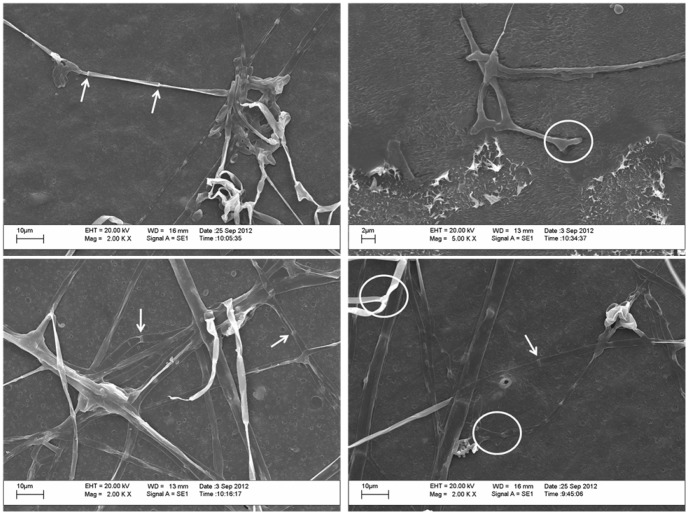
Scanning electron microscopy of *Pleurotus ostreatus* hyphae grown on the surface of oxo-biodegradable plastic. White arrows and white circles denote the septa and adhesion hyphae, respectively.

**Figure 5:**
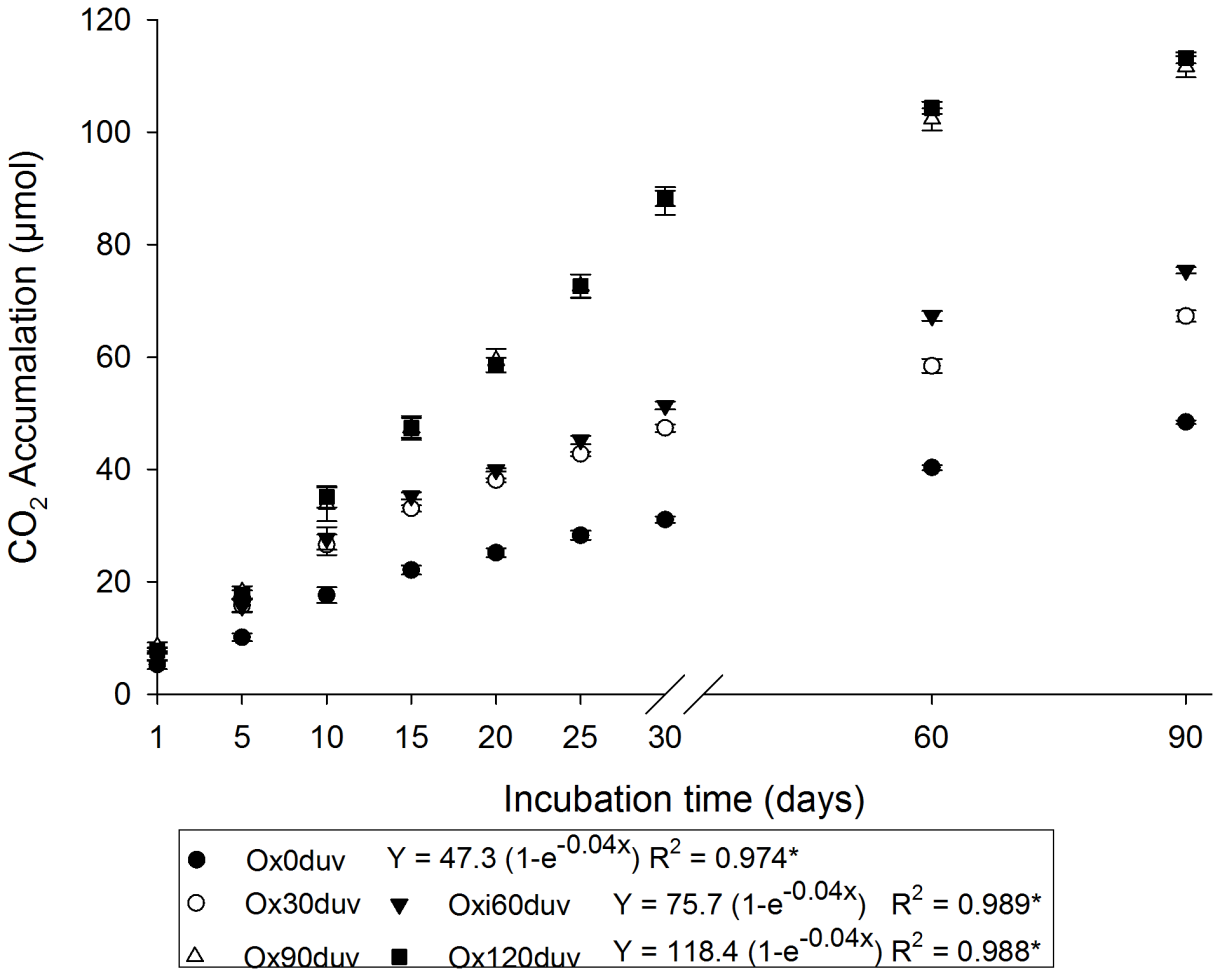
Respiratory activity of *Pleurotus ostreatus*, following 90 days of growth, before (Ox0duv) and after 30 (Ox30duv), 60 (Ox60duv), 90 (Ox90duv) and 120 days (Ox120duv) of sunlight. *Exponential equation significance (p<0.05). There were no differences between Ox30duv and Ox60duv or Ox90duv and Ox120duv.

In this study, we observed mycelial growth of *P. ostreatus*, characterized by apocytic mycelium and adhesion hyphae, on the surface of the plastic (Figure 4, Figure S2). The highest respiratory activity of *P. ostreatus* was measured after 90 or 120 days of sunlight (Figure 5). No differences were observed between samples exposed for 30 and 60 days or 90 and 120 days (Figure 5).

**Figure 6:**
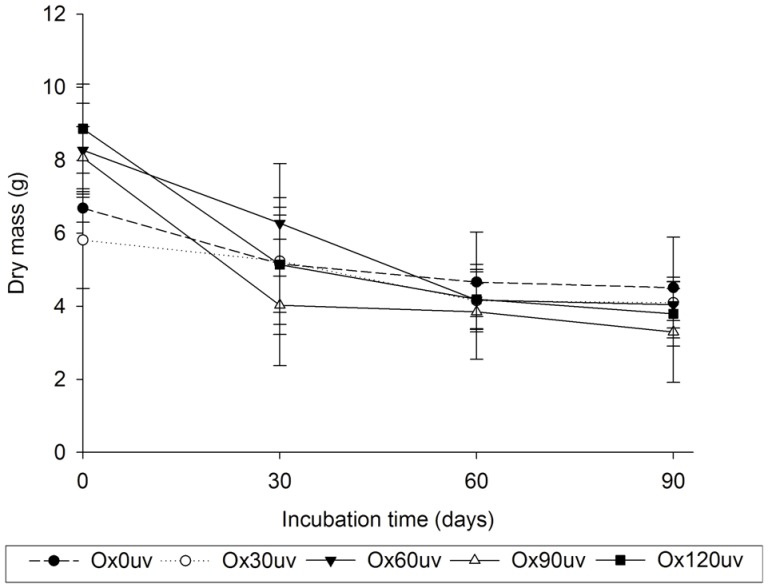
Dry mass of the substrates, used for 90 days of *Pleurotus ostreatus* growth, before (Ox0duv) and after 30 (Ox30duv), 60 (Ox60duv), 90 (Ox90duv) or 120 days (Ox120duv) of sunlight.

We observed a reduction in the dry mass of substrates during fungal incubation (Figure 6). This reduction was highest in plastics residues with over 60 days of exposure to sunlight (Figure 6). Altogether, these results show that the fungus used the substrates for metabolism and growth. Furthermore, we observed a negative correlation between respiratory activity and the dry mass of the substrates (r = −0.977).

Throughout the incubation period, we observed a deterioration of the mechanical properties of the plastic (Table 2), which was greater after 90 days. This result provides additional evidence of polymer degradation by *P*. *ostreatus*.

**Table 2:** Mechanical properties of oxo-biodegradable plastic before and after 30, 60 or 90 days of incubation with *Pleurotus ostreatus*.

Mechanical properties	Time of incubation (days)	Exposition time of the ultraviolet light (days)				
		0	30	60	90	120
Maximum load of break (N)	30	3.234±0.785	2.095±0.551	2.053±0.870	1.857±0.296	1.595±0.208
	60	2.629±0.650	1.342±0.357	1.309±0.982	1.292±0.046	1.130±0.271
	90	2.137±0.298	0.970±0.328	0.952±0.242	0.924±0.484	0.909±0.277
Energy at break (J)	30	0.064±0.018	0.052±0.011	0.034±0.006	0.032±0.007	0.022±0.007
	60	0.055±0.026	0.047±0.017	0.027±0.008	0.030±0.015	0.016±0.002
	90	0.042±0.003	0.038±0.009	0.024±0.006	0.026±0.001	0.010±0.008
Tensile extension at break (cm)	30	4.763±0.950	4.193±1.048	4.308±0.991	4.222±1.015	3.815±0.366
	60	4.230±0.432	4.128±1.004	4.101±0.482	3.564±0.704	3.497±0.542
	90	4.141±0.724	4.096±0.690	4.067±0.878	3.550±0.665	2.957±0.833
Load at tensile strength (N)	30	2.761±0.644	1.394±0.189	1.354±0.503	1.242±0.473	1.164±0.463
	60	2.352±0.873	1.170±0.876	1.230±0.632	1.218±0.687	1.102±0.510
	90	1.902±0.593	1.110±0.392	1.119±0.425	1.098±0.658	0.954±0.266
Elastic modulus (MPa)	30	27.962±4.771	18.391±3.139	16.101±2.078	14.523±0.841	13.003±1.076
	60	23.470±3.341	14.960±2.333	10.326±2.940	12.311±0.350	9.344±0.954
	90	20.190±2.965	13.639±4.350	9.854±1.724	9.730±0.831	7.922±0.712

**Figure 7:**
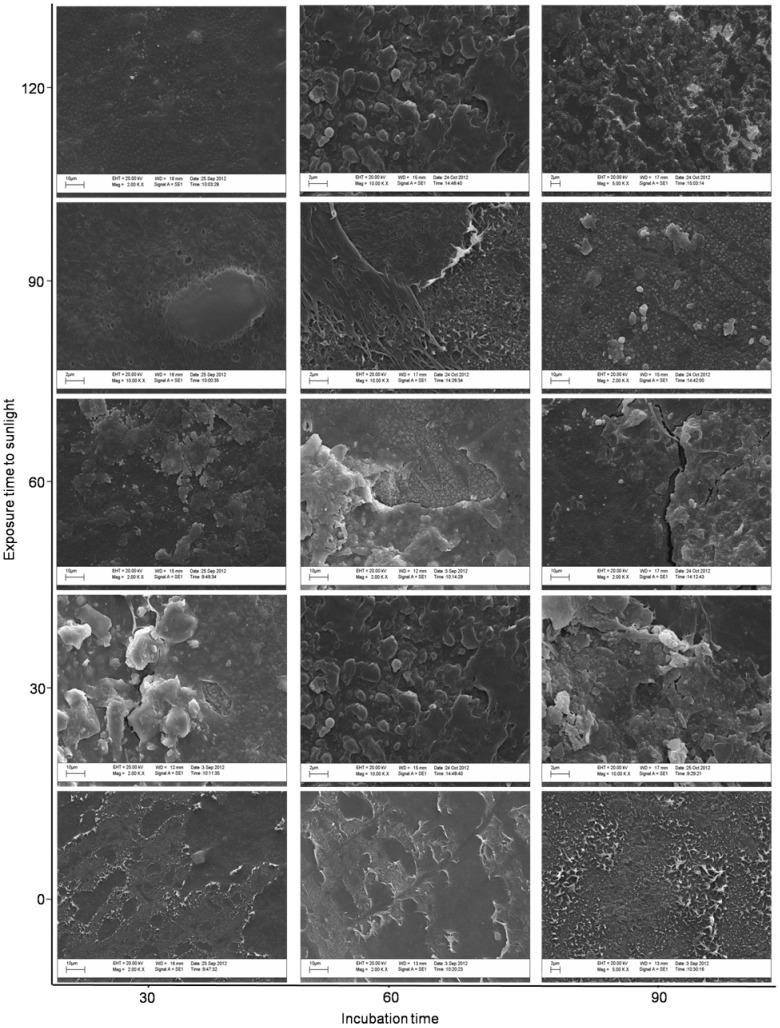
Scanning electron microscopy of oxo-biodegradable plastic before and after 120 days of sunlight and after 30, 60 and 90 days of incubation with *Pleurotus ostreatus*.

Regardless of sunlight exposure, *P. ostreatus* altered the surface of the oxo-biodegradable plastic (Figure 7). However, changes were more evident in plastic exposed to sunlight and more dramatic after longer exposure time (Figure 7). Small pits and breaks occurred after 30 days and larger cracks and fragments after 60 days. By the end of 90 days, separation of fragments and scarification were observed (Figure 7).

**Figure 8:**
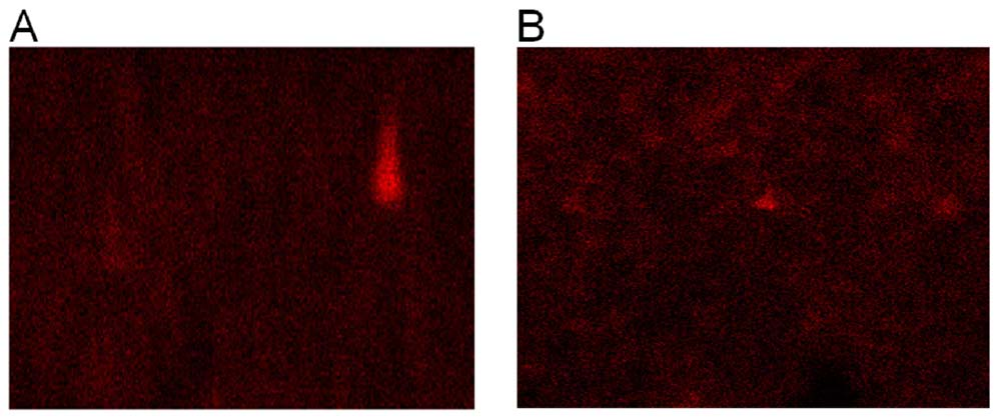
X-ray diffraction of oxo-biodegradable plastic before exposure to sunlight (A) and after 120 days of sunlight and 90 days of incubation. The small red points represent titanium.

After 210 days, including sunlight exposure and fungal incubation, we detected reduced titanium oxide concentrations on the surface of the plastic (Figure 8). This reduction is indicative of TiO_2_ catalytic oxidation, possibly initiated by sunlight and the action of fungal enzymes. Thus, titanium oxide appears important for the breakdown of polymer chains into fragments (Figure 8), which can then be used by the fungus as a carbon and energy source.

The FTIR spectrum of the plastic yielded four different bands, indicating chemical changes that occurred after incubation with *P. ostreatus* (Table 3). Although the presence of these bands was independent of sunlight exposure and time of incubation, greater exposure to sunlight during longer incubation periods led to increased band intensity (Table 3).

**Table 3:** Fourier transform infrared spectroscopy spectra of chemical alterations in oxo-biodegradable plastic after 30, 60 and 90 days of incubation with *Pleurotus ostreatus*.

Wavenumbers (cm^−1^)	band		Putative chemical groups
	Number	Intensity/type	
3500–3000	1	weak/wide	Hydroxyl (-OH)
1500–1000	3	strong/narrow	Bond carbon hydrogen (CH or CH_2_)
		weak/wide	Bond carbon oxygen (CO)
		strong/narrow	Bond of ether or peroxide

**Figure 9:**
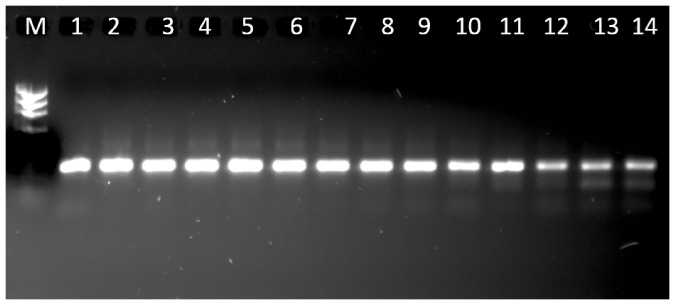
Bands of *nifH* gene from *Pleurotus ostreatus* mycelium. Numbers represent the order of the bands (see table 4). M =  marker.

*Pleurotus ostreatus* was able to grow using oxo-biodegradable plastics as a carbon and energy source. However, these plastics have a very low concentration of nitrogen (Table 1), an element required for fungal growth. Thus, the ability of *P. ostreatus* to grow on the plastic may depend on endomycotic nitrogen-fixing bacteria found in the fungal hyphae. The presence of these bacteria was confirmed by the amplification of the *nifH* gene (Figure 9). The sequence of this nitrogenase gene was similar to those from other species of nitrogen-fixing microorganisms (Table 4).

**Table 4:** Comparison of the *nifH* gene sequence from *Pleurotus ostreatus* and sequences of similar genes deposited in GenBank.

Bands*	Acess Genbank	Identification	Identity (%)	Similarity (%)
1	YP_553849.1	Nitrogenase reductase [*Burkholderia xenovorans* LB400]	93	90
2	YP_484590.1	Nitrogenase reductase [*Rhodopseudomonas palustris* HaA2	72	62
3	YP_005075592.1	Nitrogenase iron protein subunit NifH [*Paenibacillus terrae* HPL-003]	87	80
4	YP_004012288.1	Nitrogenase iron protein [*Rhodomicrobium vannielii* ATCC 17100]	82	75
5	ZP_09750647.1	Nitrogenase iron protein [*Burkholderiales* bacterium JOSHI_001]	73	71
6	YP_553849.1	Nitrogenase reductase [*Burkholderia xenovorans* LB400]	93	88
7	YP_004012288.1	Nitrogenase iron protein [*Rhodomicrobium vannielii* ATCC 17100]	97	95
8	NP_768409.1	Nitrogenase reductase [*Bradyrhizobium japonicum* USDA 110]	96	93
9	YP_004012288.1	Nitrogenase iron protein [*Rhodomicrobium vannielii* ATCC 17100]	96	88
10	ZP_09750647.1	Nitrogenase iron protein [*Burkholderiales bacterium* JOSHI_001]	97	97
11	ZP_09750647.1	Nitrogenase iron protein [*Burkholderiales bacterium* JOSHI_001]	98	95
12	ZP_09750647.1	Nitrogenase iron protein [*Burkholderiales bacterium* JOSHI_001]	97	96
13	ZP_09750647.1	Nitrogenase iron protein [*Burkholderiales bacterium* JOSHI_001]	95	93
14	ZP_09750647.1	Nitrogenase iron protein [*Burkholderiales bacterium* JOSHI_001]	97	95

*See figure 9.

## Discussion

This study, in correlation with those by da Luz et al. [9], confirm the manufacturer's claim that the plastic bags were made of oxo-biodegradable polyethylene containing titanium oxide as pro-oxidant (Table 1; Figures 1 and 2). This oxide-containing oxo-biodegradable plastics have been studied previously as well [5],[7],[17],[18].

Exposing oxo-biodegradable plastics to sunlight was not sufficient to initiate abiotic degradation (Table 1; Figures 1, 2 and 3). However, prior sunlight exposure caused modifications that spurred biotic degradation (Table 1; Figures 1, 3 and 7). According to Bonhomme et al. [12], UV light or heat is an essential precursor for biodegradation.

The fungal respiratory activities during 90 days of sunlight (Figure 5) can be explained by exponential equations (p<0.05). Regardless of the substrate, these types of exponential equations for respiratory activity of fungi are generally observed [19]–[21]. The decrease of respiratory activity at the end of incubation period is also common due to the depletion of nutrients, the accumulation of cells with a low metabolism and an increase in inhibitory metabolic compounds [22].

Pro-oxidants are incorporated into polymer chains to accelerate photo- and thermo-oxidation [7],[8]. As a result, plastics exposed to UV light or high temperatures are degraded by the interaction of free radicals with atmospheric oxygen. According to Koutny et al. [7] this interaction cleaves polymer chains into low molecular weight compounds such as carboxylic acids, alcohols and ketones. However, their study exposed polymers to artificial UV light, whereas we simulated plastic degradation under more natural conditions. Furthermore, we detected reduced titanium oxide concentrations on the surface of the plastic (Table 1), evidence of TiO_2_ photocatalytic oxidation. We also found that sunlight did not cause formation of new functional groups (Figure 2), cracks or pits (Figure 3). Thus, the use of different methodologies or variability in samples obtained from different manufacturers may account for the different results obtained by our study compared to those of Koutny et al. [7].

Although da Luz et al. [9] showed that *P. ostreatus* degrades oxo-biodegradable plastic without any prior physical treatment, we found that sunlight modified the plastic's mechanical properties and reduced its titanium oxide concentration (Table 1, Figure 1), both of which were important for fungal growth and biodegradation (Figures 4, 5 and 7). Incorporation of titanium oxide within the polymer chains (Table 1, Figure 8), the low specificity of the fungal lignocellulolytic enzymes for their substrates and the presence of endomycotic nitrogen-fixing microorganisms (Figure 9, Table 4) may have also enhanced the degradation of the plastic by *P. ostreatus*. Fungal growth using oxo-biodegradable plastics as a carbon source has been previously shown [5],[7],[9],[23],[24].

The fragmentation of the polyethylene polymer chain can be observed by the formation of band of carbon-hydrogen bond (Table 3). The other bands observed indicate that oxidation occurred in this chain (Table 3). Thus, the fragmentation of polyethylene may have been due to oxidation dependent or independent of the titanium oxide. This oxide-independent oxidation may be due to the activity of fungal enzymes during the incubation or to the formation of free radicals by *P. ostreatus*. Oxygen-hydrogen bonds and hydrogen peroxide can also form during photocatalytic oxidation [8].

Lignocellulolytic enzymes degrade lignocellulosic residues and various pollutants and recalcitrant compounds, such as polycyclic aromatic hydrocarbons [25], trinitrotoluene [26], phorbol ester [27] and phytate [28]. The high variety of compounds degraded by lignocellulolytic enzymes, many of which bear little or no structural similarity to the more favored substrates, highlights the enzymes' low specificity. Thus, degradation of oxo-biodegradable plastic polymers may occur due to the action of this group of enzymes in a co-metabolic process. Co-metabolic processes are commonly used in the treatment of recalcitrant compounds, where a carbon source is added to induce the synthesis of certain enzymes that degrade not only natural substrates but also undesired pollutants or residues [29],[30]. As in our previous work, paper towel provided the carbon source that initiated fungal growth and synthesis of lignocellulolytic enzymes [9]. Chemical and physical alterations to the plastic after sunlight exposure (Table 1, Figure 1) may have intensified enzymatic activity (Figure 7). Sivan [31] and Santo et al. [32] suggest that laccases have an important role in the biodegradation of oxo-biodegradable polyethylene.

Although nitrogen is essential for fungal metabolism, we have previously found that *P. ostreatus* grows on substrates with high carbon to nitrogen ratios [9],[10],[29],[33]. Thus, we hypothesized that this fungus has a symbiotic relationship with nitrogen-fixing microorganisms. Here, we sequenced the *nifH* gene in *P. ostreatus* PLO6 (Figure 9, Table 4). The presence of this gene in *P. ostreatus* [34] and other basidiomycetes [35] implies an association with endosymbiotic nitrogen-fixing bacteria. Nitrogen-fixing microorganisms in the fungal hyphae may supply the nitrogen necessary for fungal metabolism. Thus, when nitrogen levels are low, the fungus would still be able to grow and synthesize enzymes that degrade primarily carbon- and hydrogen-containing substrates such as lignin, cellulose, polycyclic aromatic hydrocarbons and oxo-biodegradable polyethylene. In our study, endomycotic nitrogen-fixing microorganisms may have facilitated fungus growth on the nitrogen-poor oxo-biodegradable plastic. Interactions between nitrogen-fixing microorganisms and *P. ostreatus* also reportedly play a key role in bio-bleaching and bioremediation processes [34].

Although we showed that *P. ostreatus* degraded oxo-biodegradable plastic (Figure 7) and reduced the dry mass of the substrates (Figure 6), mineralization of the samples was not complete. After 210 days of physical and biological treatments, plastic fragments colonized by fungus still remained in the flask. Since degradation has already begun, composting the remaining material would complete the biodegradation process. Esmaeili et al. [23] showed that Aspergillus sp. and Lysinibacillus sp., both isolated from landfill soil, degrade low-density polyethylene.

## Conclusions

The degradation of oxo-biodegradable plastics occurs by abiotic and biotic reactions. Exposing plastics to sunlight stimulates abiotic reactions, which, in turn, fuel biotic reactions driven by lignocellulolytic enzymes. The presence of a pro-oxidant is also important for abiotic degradation. Likewise, endomycotic nitrogen-fixing bacteria facilitate the growth of fungus on this nitrogen-poor substrate and, consequently, stimulate plastic biodegradation.

## Supporting Information

Figure S1 **DGGE analysis of the** ***nifH*** **gene from different microorganisms and** ***Pleurotus ostreatus*** **mycelium.** Numbers represent the order and amount of bands (see table 4).
(TIF)

Figure S2 **Mycelial growth of** ***Pleurotus ostreatus*** **during 30, 60 and 90 days of incubation in oxo-biodegradable plastics that were exposured for 0, 30, 60, 90 or 120 days to sunlight.**
(TIF)
