# Supplementary figures and images for: Abiotic and Biotic Degradation of Oxo-Biodegradable Plastic Bags by Pleurotus ostreatus
Source: PLoS One. 2014 Nov 24;9(11):e107438. doi: 10.1371/journal.pone.0107438 (PMC4242504; doi:10.1371/journal.pone.0107438)

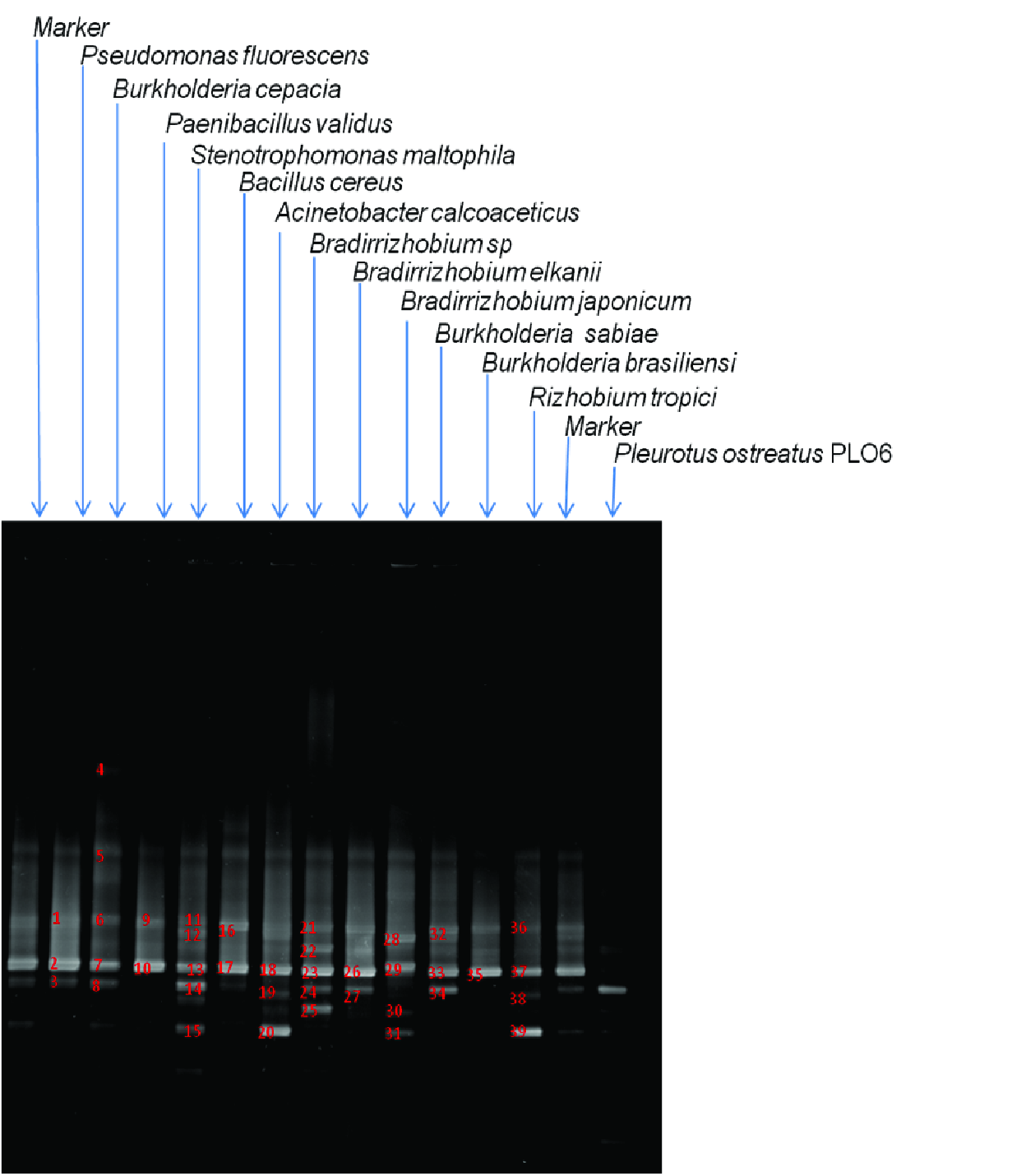

Supplement: Figure S1 — DGGE analysis of the nifH gene from different microorganisms and Pleurotus ostreatus mycelium. Numbers represent the order and amount of bands (see table 4). (TIF) [file pone.0107438.s001.tif]

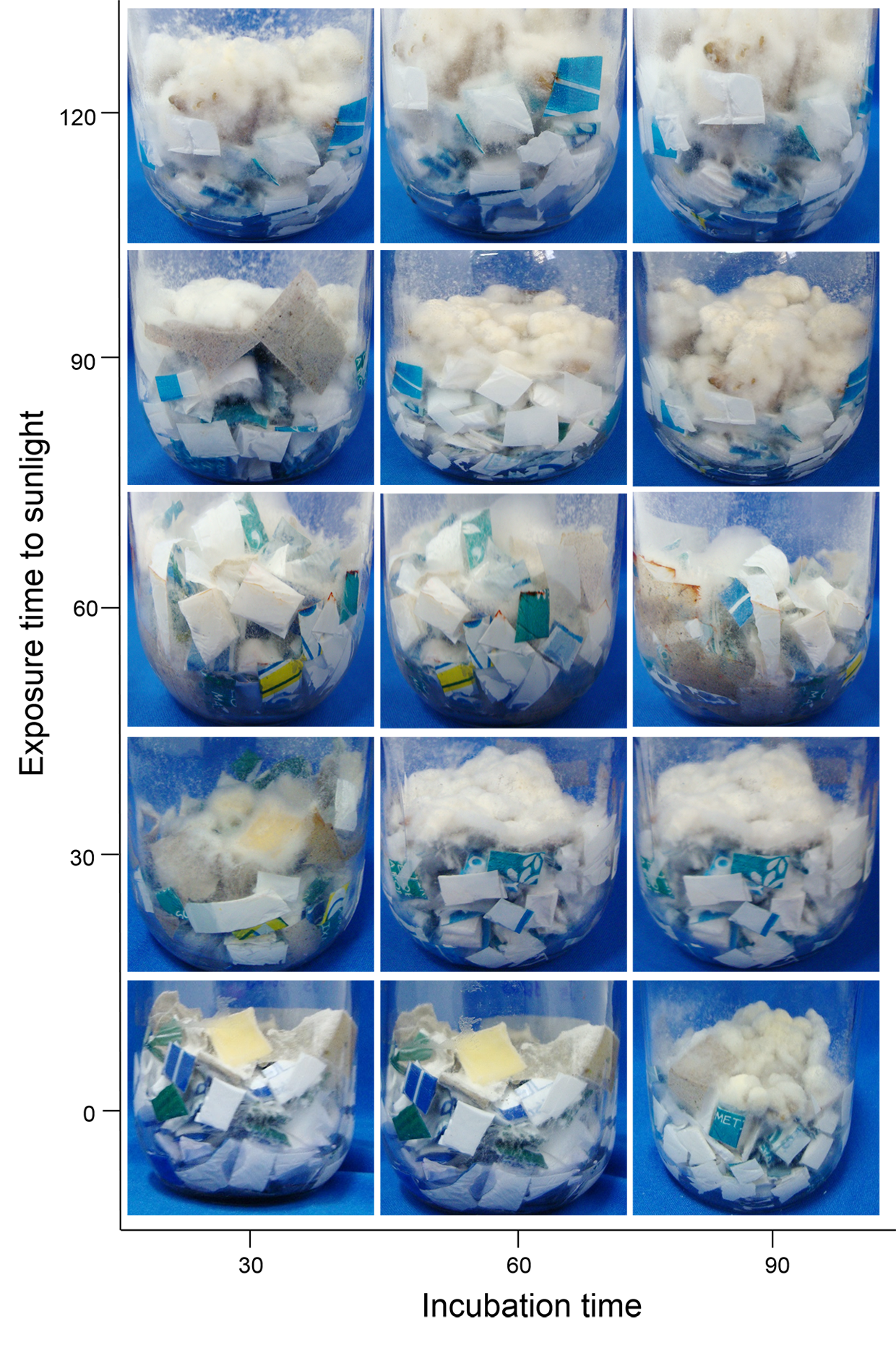

Supplement: Figure S2 — Mycelial growth of Pleurotus ostreatus during 30, 60 and 90 days of incubation in oxo-biodegradable plastics that were exposured for 0, 30, 60, 90 or 120 days to sunlight. (TIF) [file pone.0107438.s002.tif]
